# Beneficial effects of *Hibiscus rosa-sinensis* L. flower aqueous extract in pregnant rats with diabetes

**DOI:** 10.1371/journal.pone.0179785

**Published:** 2017-06-23

**Authors:** Luana Alves Freitas Afiune, Thaís Leal-Silva, Yuri Karen Sinzato, Rafaianne Queiroz Moraes-Souza, Thaigra Sousa Soares, Kleber Eduardo Campos, Ricardo Toshio Fujiwara, Emilio Herrera, Débora Cristina Damasceno, Gustavo Tadeu Volpato

**Affiliations:** 1 Laboratory of System Physiology and Reproductive Toxicology, Institute of Biological and Health Sciences, Federal University of Mato Grosso (UFMT), Barra do Garças, Mato Grosso State, Brazil; 2 Gynecology, Obstetrics and Mastology Graduate Course, Laboratory of Experimental Research on Gynecology and Obstetrics, Botucatu Medical School, Univ Estadual Paulista_Unesp, Botucatu, São Paulo State, Brazil; 3 Department of Parasitology, Institute of Biological Sciences, Federal University of Minas Gerais (UFMG), Belo Horizonte, Minas Gerais State, Brazil; 4 Department of Biochemistry and Molecular Biology, University CEU San Pablo, Madrid, Spain; China Medical University, TAIWAN

## Abstract

**Purpose:**

The *Hibiscus rosa-sinensis* flower is widely used in Brazilian traditional medicine for the treatment of diabetes and has shown antifertility activity in female Wistar rats. However, there is no scientific confirmation of its effect on diabetes and pregnancy. The aim of this study was evaluate the effect of aqueous extract of *H*. *rosa-sinensis* flowers on maternal-fetal outcome in pregnant rats with diabetes.

**Methods:**

Diabetes was induced by streptozotocin (STZ, 40 mg/kg) in virgin, adult, female Wistar rats. After diabetes induction, the rats were mated. The pregnant rats were distributed into four groups (n minimum = 11 animals/group): non-diabetic, non-diabetic treated, diabetic, and diabetic treated. Oral aqueous extract of *Hibiscus rosa-sinensis* was administered to rats in the treatment groups during pregnancy. At term pregnancy, maternal reproductive outcomes, fetal parameters, and biochemical parameters were analyzed.

**Results:**

The non-diabetic treated group showed decreased high density lipoprotein cholesterol, increased atherogenic index (AI) and coronary artery risk index (CRI), and increased preimplantation loss rate compared to the non-diabetic group. Although treatment with *H*. *rosa-sinensis* led to no toxicity, it showed deleterious effects on cardiac and reproductive functions. However, the diabetic treated group showed increased maternal and fetal weights, reduced AI and CRI, and reduced preimplantation loss rate compared to the untreated diabetic group.

**Conclusion:**

Our results demonstrate beneficial effects of this flower only in pregnant rats with diabetes and their offspring. Although these findings cannot be extrapolated to human clinical use, they show that the indiscriminate intake of *H*. *rosa-sinensis* may be harmful to healthy individuals and its use should be completely avoided in pregnancy.

## Introduction

Several plant species are recognized in folk medicine to have antidiabetic properties [1]; however, few plants have received appropriate scientific investigation. The genus *Hibiscus* is used empirically due to possible hypoglycemic or antidiabetic effects. For this reason, many species of genus *Hibiscus* (Malvaceae) have gained the attention of researchers in recent years. *H*. *rosa-sinensis* Linn. is widely found in Brazil and is used for urban landscaping, as an ornamental plant and living fence; it is known as rose mallow, Chinese hibiscus, China rose, and shoe flower. Pharmacological studies showed that flowers of *H*. *rosa-sinensis* have numerous actions including antibacterial [2–3], wound healing [4–5], antidepressant [6–7], cardiac [8], and antioxidant [9] effects.

The usage of plant-derived antioxidants is considered as an alternative strategy for improving oxidative damage in diabetes. There is evidence that hyperglycemia-mediated oxidative stress induces free radical generation, which leads to maternal complications [10–12] and fetal distress [13–14]. The extract of flowers of *H*. *rosa-sinensis* contains phenolic compounds and flavonoids responsible for its antioxidant activity [15]. In addition, the flowers and leaves of this plant showed significant hypoglycemic effect in several studies [16–19]. However, there are few studies related to diabetes, pregnancy and treatment with medicinal plants [20], and no studies evaluating the effect of *Hibiscus rosa-sinensis* in diabetic pregnancy.

We hypothesized that *H*. *rosa-sinensis* would have a beneficial effect in diabetic pregnancy without deleterious effects on the mother or the fetus. Therefore, the aim of this study was to evaluate the effect of aqueous extract of *H*. *rosa-sinensis* flowers on pregnant rats with diabetes and to investigate maternal reproductive and fetal outcomes.

## Materials and methods

### Extraction of plant materials

Flowers of *H*. *rosa-sinensis* were collected from Barra do Garças, Mato Grosso State, Brazil, between May and June 2012, in the morning. The plant was identified and authenticated by the Instituto Nacional de Ciência e Tecnologia—Herbário Virtual da Flora e dos Fungos (INCT-HVFF), where a voucher specimen (157459) was left. The flowers of the plant were dried at 35 ^o^C for a period of 24 h in an aerated stove, ground, and a powder was prepared. *H*. *rosa-sinensis* aqueous extract was prepared by boiling 20 g of the flower powder in 1 L of water for 5 min. The extract was agitated and covered until it reached room temperature. The residue was removed by filtration (1 mm pore size) and the extract was then adequately concentrated in a rotary evaporator (1 hour). A sample was separated for determination of the solid concentration, and the extract was divided into aliquots stored at -20^°^C until further use.

### Experimental animals

Female Wistar rats (230–250 g) were obtained from the Federal University of Mato Grosso Vivarium and were maintained under standard laboratory conditions (22±3 ^o^C, 12 h light/dark cycle), with pelleted food (Purina rat chow, Purina^®^, São Paulo, SP, Brazil) and tap water *ad libitum*. The procedures and animal handling were authorized by the Ethical Committee for Animal Research of the UFMT, Brazil (Protocol number 23108.001991/13-1).

After two weeks of acclimatization, diabetes was induced in rats with streptozotocin (STZ; Sigma Chemical Company^®^, St. Louis, Millstone). STZ was intravenously (i.v.) administered at a single dose of 40 mg/kg dissolved in citrate buffer (0.1 M, pH 6.5). Non-diabetic rats received i.v. citrate buffer. Tail blood glucose concentrations were measured using a One Touch Ultra glucometer (Johnson & Johnson^®^) 7 days after the STZ treatment and glucose concentrations exceeding 300 mg/dL confirmed the presence of diabetes [21].

### Experimental groups

After one week of confirmation of diabetic state, virgin female Wistar rats were mated overnight with non-diabetic male Wistar rats. The morning on which spermatozoids were found in the vaginal smear (day 0 of pregnancy), the rats were divided at random into four experimental groups: non-diabetic (n = 12), non-diabetic treated with *H*. *rosa-sinensis* extract (n = 13), diabetic (n = 12), and diabetic treated with *H*. *rosa-sinensis* extract (n = 11). Treatments were given orally, by *gavage*, in the morning. An initial dose of 100 mg/kg/day of the *H*. *rosa-sinensis* extract was given from day 0 until the 7th day of pregnancy (implantation period). The dose was increased to 200 mg/kg/day from day 8 to 14 of pregnancy (embryonic period), and to 400 mg/kg/day from day 15 to 20 (fetal period).

### Course of pregnancy

Glycemia was measured in tail blood every seven days up to the end of pregnancy, at approximately 9 a.m. At days 0 and 21 of pregnancy, body weight gain, food consumption, and water intake were measured. At day 21 of pregnancy, rats were anesthetized by sodium thiopental (Thiopentax^®^, São Paulo, Brazil—50 mg/kg body weight, intraperitonialy) and after decapitation, blood samples were collected from the neck wound for biochemical analysis. Liver tissue was collected and processed as described below to determine oxidative stress markers. The gravid uterus was weighed and dissected to count dead and live fetuses, resorption, implantation, and corpora lutea numbers. The number of implantation sites was determined by the Salewski method [22]. The rate of pre-implantation loss was calculated as: (number of corpora lutea—number of implantations) × 100 / number of corpora lutea. Post-implantation loss rate was calculated as: (number of implantations—number of live fetuses) ×100 / number of implantations [23].

The fetuses were weighed and classified as small (SPA), adequate (APA), or large (LPA) for pregnancy age [24], and evaluated microscopically to determine the presence of external anomalies [23]. After external analysis, half the fetuses were fixed in Bouin’s fluid and serial sections were prepared as described by Wilson [25] for visceral examination. The remaining fetuses were prepared for examination of the skeletons by the staining procedure of Staples and Schnell [26].

### Biochemical profile analysis

The collected blood samples were were collected in dry tubes and maintained on ice for 30 min and then centrifuged at 1300 *g* for 10 min at 4°C. The serum supernatant was at -80°C for determination of biochemical parameters.

Serum concentrations of total cholesterol (CHO), triglycerides (TG), and high-density lipoprotein (HDL) concentrations were estimated by enzymatic methods [27] using Winner^®^ assay kits (Rosário, Argentina). Very-low-density lipoprotein (VLDL) cholesterol values were calculated from the triglyceride concentrations [28]. The atherogenic index (AI) was calculated according to the method of Liu *et al*. [29] and expressed as: AI = (CHO-HDL)/HDL. Coronary artery risk index (CRI) was also calculated by the following formula: CHO/HDL [30]. A standard line were used the values of control group.

### Oxidative stress markers in the liver

Collected liver samples were rapidly washed with phosphate buffer saline (0.01 M, NaCl 0.138 M, KCl 0.0027M, pH 7.4). Hepatic malondialdehyde (MDA) and total glutathione (GSH-t) level and superoxide dismutase (SOD) activity were determined using commercial kits (Cayman^®^ Chemical Co., Ann Arbor, Michigan, U.S.A.).

Catalase (CAT) activity was determined following decreases in the initial H_2_O_2_ concentration (20 nM used as the initial substrate) at 240 nm and 25 ^o^C, over a time frame of 120 seconds. Briefly, 1 μL of supernatant isolated from rat liver homogenates was diluted in phosphate-buffered saline (0.1 M, pH 7). H_2_O_2_ 1 M was added to obtain a final volume of 1 mL with H_2_O_2_ 2 mM in a cuvette. The decrease in absorption at 240 nm in 120 seconds was determined and CAT activity was calculated using a molar extinction coefficient (ɛ = 0.0436 nM/cm) and expressed as U/mg protein [31].

Reduced thiol group levels in liver homogenates were evaluated by the method described by Jollow *et al*. [32] based on the development of a yellow color when 5,5’-Dithiobis(2-nitrobenzoic acid) (DTNB) was added to compounds containing sulfhydryl groups. The absorbance was read at 412 nm.

### Statistical evaluation

We acknowledge that the homogeneity among experimental units is one of the basics of experimental design, and considering that non-diabetic and diabetic treated rats are biologically different organisms. Thus, we performed the following comparisons: non-diabetic treated with *H*. *rosa-sinensis vs*. non-diabetic groups, non-diabetic *vs*. diabetic groups, diabetic treated with *H*. *rosa-sinensis vs*. non-diabetic treated groups, and diabetic treated *vs*. diabetic groups. Student's unpaired t-test for normal distribution and Mann-Whitney U test for abnormal distribution of data were used to compare two groups. The proportion data were analyzed by Fisher's exact test. *p* < 0.05 was considered statistically significant.

## Results

**Fig 1:**
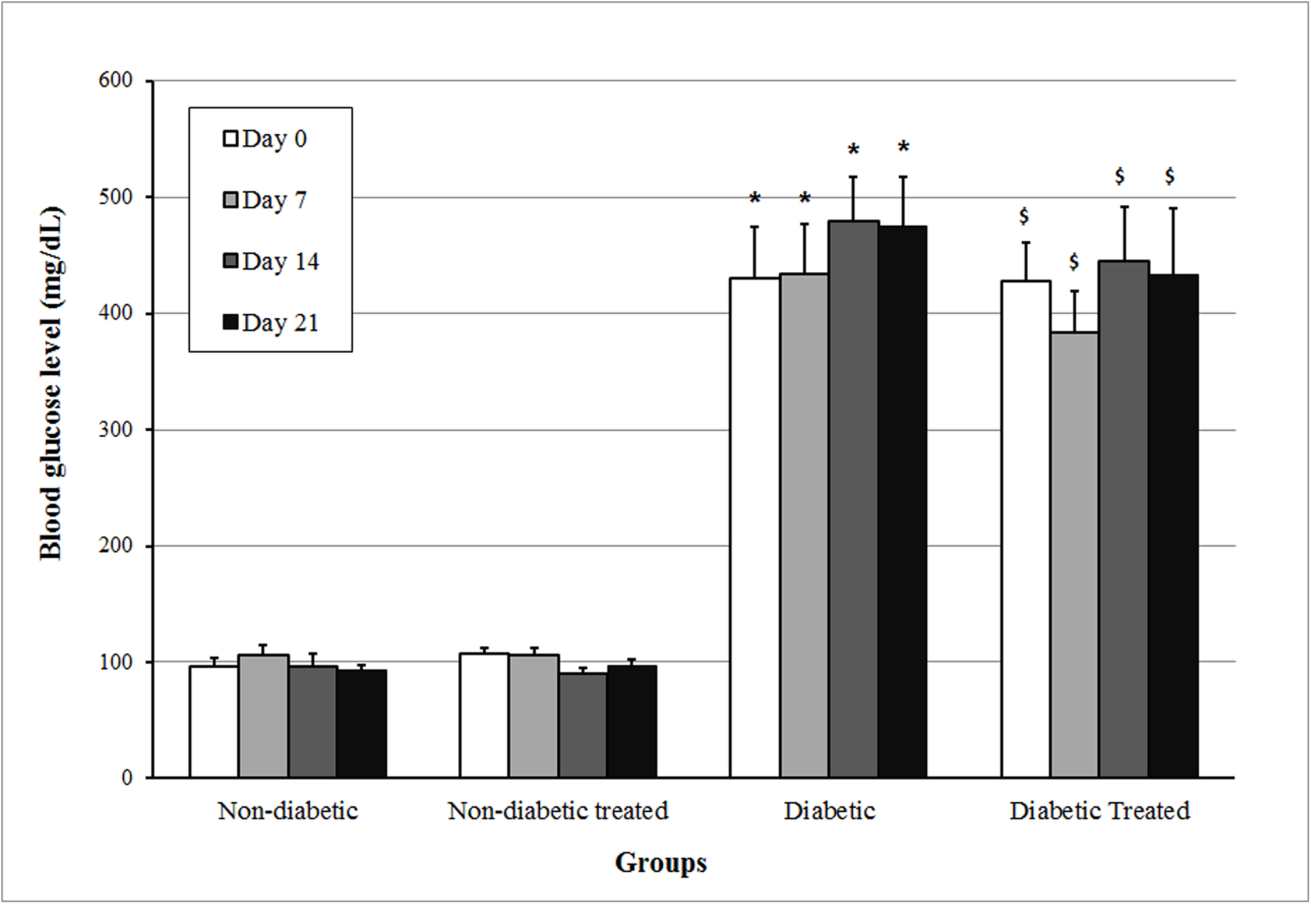
Blood glucose level on days 0, 7, 14, and 21 of non-diabetic and diabetic rats not treated or treated with *Hibiscus rosa-sinensis* aqueous extract during pregnancy. N minimum = 11 animals/group. Data shown as mean ± standard error. *p < 0.05 compared to non-diabetic group (t test); ^$^p < 0.05 compared to non-diabetic treated group (Mann-Whitney test).

As shown in Fig 1, blood glucose levels of the rats of the two non-diabetic groups remained at approximately 100 mg/dL throughout the 21 days of pregnancy. In the diabetic groups, blood glucose levels were always above 300 mg/dL and treatment with the aqueous extract of *Hibiscus rosa-sinensis* did not modify blood glucose levels in non-diabetic or diabetic rats.

**Fig 2:**
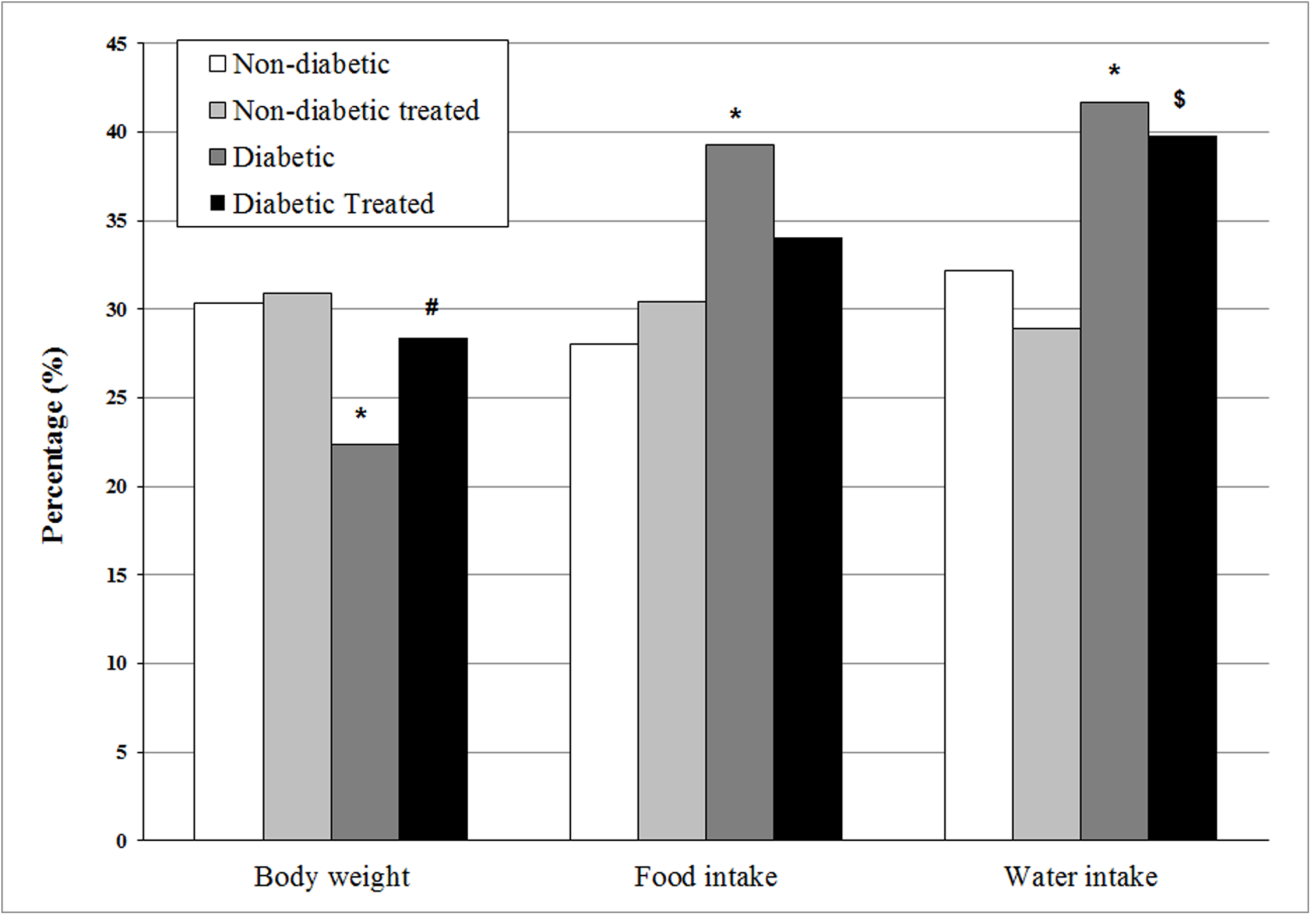
Percentage of body weight gain, food intake, and water intake at 21 days of pregnancy as compared to onset of pregnancy in non-diabetic and diabetic rats not treated or treated with *Hibiscus rosa-sinensis* aqueous extract during pregnancy. N minimum = 11 animals/group. *p < 0.05 compared to non-diabetic group; ^#^p < 0.05 compared to diabetic group; ^$^p < 0.05 compared to non-diabetic treated group (Fisher exact test).

Treatment did not change body weight, water intake, or food intake in non-diabetic rats. The diabetic group not receiving treatment showed decreased body weight and increased food and water intake compared to the non-diabetic group. With the exception of the high water intake, the diabetic treated rats showed normalization of body weight and food intake to values that did not differ from those in the non-diabetic group (Fig 2).

Biochemical profiles are shown in Table 1. In non-diabetic rats, treatment decreased HDL concentration and increased both the AI and CRI compared to rats not receiving treatment. The diabetic rats not receiving treatment had higher TG, CHO, VLDL, and MDA concentrations, and higher AI and CRI compared to the non-diabetic rats. In addition, the HDL concentration was lower in diabetic rats than in non-diabetic rats. There was a significant increase in MDA concentration and SOD activity in diabetic treated rats compared to non-diabetic treated rats. Moreover, the diabetic treated rats showed decreased TG and VLDL concentrations, increased HDL concentration, and decreased AI and CRI compared to diabetic untreated animals.

**Table 1:** Biochemical profile at 21 days of pregnancy in non-diabetic and diabetic rats not treated or treated with *Hibiscus rosa-sinensis* aqueous extract during pregnancy.

	Groups			
	*Non-diabetic* *(n = 12)*	*Non-diabetic treated (n = 13)*	*Diabetic* *(n = 12)*	*Diabetic treated (n = 11)*
TG (mg/dL)	136.1 ± 14.6	195.3 ± 33.6	591.3 ± 53.3*	334.1 ± 104.1^#^
CHO (mg/dL)	81.3 ± 1.8	114.6 ± 15.4	143.5 ± 12.3*	111.6 ± 9.2
HDL-c (mg/dL)	43.7 ± 2.0	24.0 ± 2.4*	15.3 ± 1.4*	22.4 ± 1.2^#^
VLDL-c (mg/dL)	27.2 ± 2.9	39.1 ± 6.7	118.3 ± 10.6*	66.8 ± 20.7^#^
AI	1.0 ± 0.1	4.3 ± 0.9*	8.6 ± 1.4*	4.6 ± 0.7^#^
CRI	2.0 ± 0.1	5.3 ± 0.9*	9.8 ± 1.5*	5.6 ± 0.7^#^
MDA (nM/mg protein)	92.2 ± 3.0	88.2 ± 10.3	214.5 ± 8.6*	183.5 ± 13.4^$^
SOD (U/mg protein)	4.6 ± 0.3	4.5 ± 0.4	4.9 ± 0.4	6.4 ±0.6^$^
GSH-t (U/mg protein)	13.1 ± 0.5	16.6 ± 0.5	16.7 ± 1.2	15.9 ± 1.1
Thiol groups (mM/mg protein)	1.2 ± 0.1	1.4 ± 0.1	1.2 ± 0.1	1.1 ± 0.1
CAT (U/mg protein)	6.5 ± 0.4	7.6 ± 1.3	4.5 ± 0.5	4.0 ± 0.7

Data shown as mean ± standard error (SD).

*p < 0.05 compared to non-diabetic group

^#^p < 0.05 compared to diabetic group

^$^p < 0.05 compared to non-diabetic treated group (t test).

**Fig 3:**
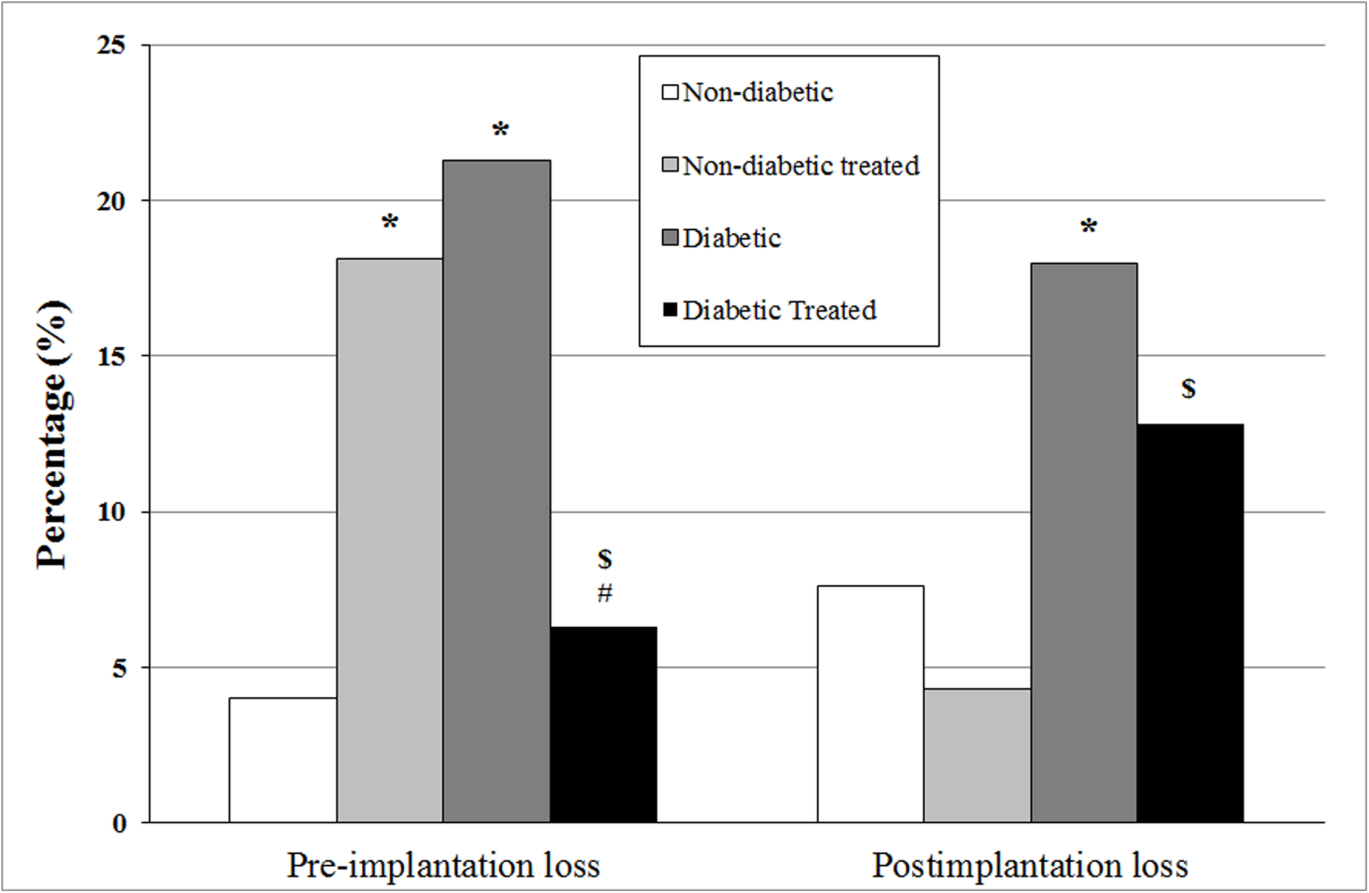
Percentage of pre- and postimplantation loss at 21 days of pregnancy in pregnant non-diabetic and diabetic rats not treated or treated with *Hibiscus rosa-sinensis* aqueous extract during pregnancy. N minimum = 11 animals/group. *p < 0.05 compared to non-diabetic group; ^#^p < 0.05 compared to diabetic group; ^$^p < 0.05 compared to non-diabetic treated group (Fisher exact test).

As shown in Fig 3, treatment increased pre-implantation losses in the non-diabetic treated group compared to the untreated group. The untreated diabetic rats showed a higher percentage of pre-implantation loses compared to non-diabetic rats. Treatment greatly decreased implantation loss in diabetic rats to values that did not differ from those in the non-diabetic group. Treatment did not modify postimplantation loss in non-diabetic rats. Untreated diabetic rats showed higher postimplantation loss than non-diabetic rats. Treatment decreased post-implantation losses in diabetics rats, although values remained higher than those in non-diabetic treated rats (Fig 3).

**Fig 4:**
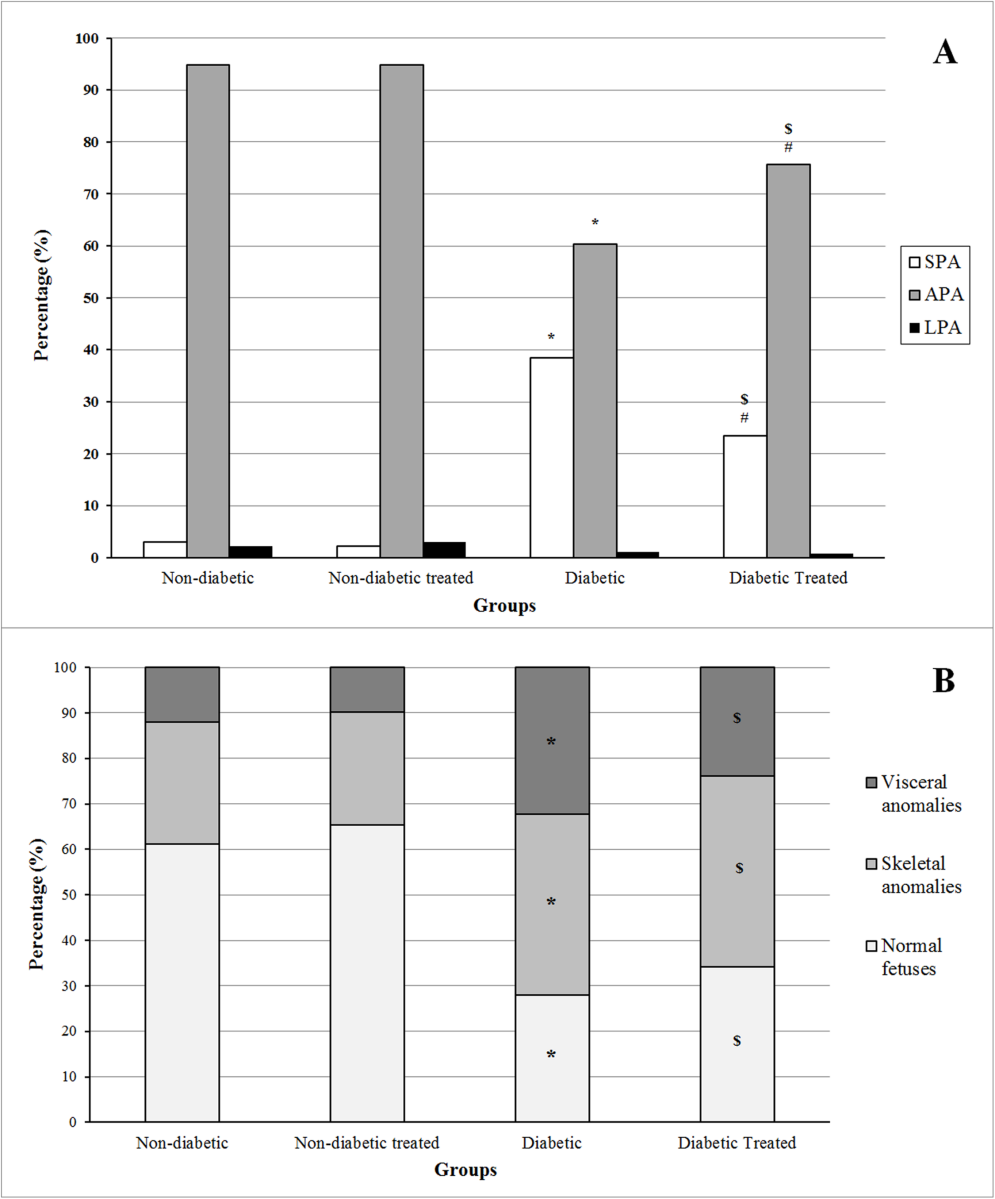
**Percentage of fetuses classified as small (SPA), adequate (APA), or large (LPA) for pregnancy age (A) and percentage of fetal anomalies (B) at 21 days of pregnancy in pregnant non-diabetic and diabetic rats not treated or treated with *H*. *rosa-sinensis* aqueous extract during pregnancy.** *Non-diabetic (n fetuses = 134); Non-diabetic Treated (n fetuses = 134); Diabetic (n fetuses = 93); Diabetic Treated (n fetuses = 117)*. *p < 0.*05 compared to non-diabetic group;* ^*#*^*p < 0*.*05 compared to diabetic group;* ^*$*^*p < 0*.*05 compared to non-diabetic treated group (Fisher exact test)*.

As shown in Fig 4A, treatment in non-diabetic rats did not modify the proportion of SPA, APA, or LPA. fetuses. The diabetic groups showed a decrease in the proportion of fetuses classified as APA and an increased proportion of fetuses classified as SPA compared to non-diabetic groups. The diabetic treated group showed an increase in the percentage of APA fetuses and a decrease in the percentage of SPA fetuses compared to the untreated diabetic group, although values did not reach those of either treated or untreated non-diabetic groups. As shown in Fig 4B, treatment did not affect the percentage of fetuses with skeletal and visceral anomalies, with a higher percentage of normal fetuses in the non-diabetic groups. However, in both the untreated and treated diabetic rats, the proportion of fetuses with visceral or skeletal anomalies was higher and the proportion of normal fetuses much lower compared to non-diabetic rats, without significant difference in values between the two diabetic groups.

## Discussion

Our results show that treatment with aqueous extract of *H*. *rosa-sinensis* flowers has no effect on blood glucose levels in non-diabetic and STZ-induced pregnant rats with severe diabetes. Others studies have shown a hypoglycemic effect of *H*. *rosa-sinensis* based on studies on traditional medicine, although these studies used a leaf aqueous extract [18–19] and flower ethanol extract [16–17,33] in non-pregnant rats. The absence of a hypoglycemic effect of *H*. *rosa-sinensis* in our study may be also related to the severity of diabetes. In fact, in the work by Venkatesh *et al*. [33], they used alloxan to induce diabetes, causing a more moderate increase in blood glucose levels than in our STZ-diabetic rats. Rats with severe diabetes induced by STZ in adult life reproduce blood glucose levels similar to that of humans with uncontrolled type 1 diabetes mellitus. The plant extract was insufficient to reduce persistent hyperglycemia in this study; this suggests that treatment in rats with moderate hyperglycemia might lead to beneficial results. Other factors that could explain the absence of a hypoglycemic effect in this study include the short treatment period, plant collect period, and the difference in sensitivity considering gender of animals tested.

Signs of uncontrolled hyperglycemia are polyphagia, polyuria, insulin deficiency, polydipsia, reduced anabolic processes, and accelerated catabolic processes. In addition, severe diabetes correlates directly with body weight loss, which is one of the most common signs of diabetes [34]; this is in agreement with our findings. We found that treatment of diabetic animals increased body weight when compared to diabetic rats, clearly showing a beneficial effect of *H*. *rosa-sinensis* extract.

*H*. *rosa-sinensis* treatment of non-diabetic pregnant rats led to reduced HDL levels and subsequent increased AI and CRI, contributing to an increased cardiovascular risk. This finding could be related to plant extract-derived nutrient overload and reticulum stress. Endoplasmic reticulum stress leads to a marked reduction of hepatic ATP-binding cassette transporter (ABCA1) expression, also known as the cholesterol efflux regulatory protein (CERP), which is a major regulator of cellular cholesterol and phospholipid homeostasis [35]. Subsequently, this reduction causes decreased HDL levels. The removal of excess cholesterol from macrophage foam cells by HDL and its principal apolipoprotein, apoA-1, is thought to be one of the key mechanisms underlying the atheroprotective properties of HDL [36–37]. Finally, this fact corroborates the negative correlation between HDL levels and AI and between HDL levels and CRI found in our study.

In contrast to non-diabetic rats, diabetic rats treated with the *H*. *rosa-sinensis* extract showed improvements in the lipid profile (reduced TG levels and increased HDL levels) and reduced cardiovascular risk, indicating a beneficial effect of the plant extract. The decrease in blood TG levels produced with treatment in the diabetic rats could contribute to this effect, since TG are known to play a regulatory role in lipoprotein interactions, rather than being an independent risk marker. This is supported by evidence that an increased plasma concentration of TG is associated with an elevated rate of coronary artery disease, increased low density lipoprotein (LDL) level, and increased cholesteryl ester transfer (HDL to apolipoprotein B) [38–39]. TG has also been proposed to be a major determinant of cholesterol esterification/transfer and HDL remodeling in human plasma [40–41]. The treatment with *H*. *rosa-sinensis* aqueous extract in diabetic male rats also showed an increased HDL-c level [4] as observed in our study. The main components of *Hibiscus rosa-sinensis* flowers extract related to antioxidant activities are tannin and anthocyanin [42]. The increase of HDL-c might be related to the anthocyanin action, since studies with anthocyanin supplementation in animals and humans showed similar results with improvement in the atherogenic activity, which was attributed to the action on reverse cholesterol transport [43–44]. However, the mechanism by which anthocyanins act is not well established.

There is evidence that STZ-induced diabetes promotes increased lipid peroxidation and decreased antioxidant enzymatic status, contributing to oxidative stress [45–47]. In our study, treatment with the plant extract in diabetic rats caused increased SOD activity, but this increase is not sufficient to influence non-diabetic oxidative stress induced by hyperglycemia, as shown by the unchanged elevated levels of MDA. Studies with *Hibiscus sabdariffa* aqueous extract showed anthocyanin-derived compounds presented high antioxidant capacity, with increased SOD activity in liver [48–49]. However, anthocyanin may have worse interaction with peroxyl radicals, the main radical species generate in the TBARS assay [50], and maybe this fact the MDA levels showed no difference in our study.

*H*. *rosa-sinensis* extract led to lipid metabolic perturbations in non-diabetic rats, which probably contributed to an inadequate intrauterine environment during embryonic implantation, making embryo attachment more difficult. This is characterized by an increased preimplantation loss rate, evident in our non-diabetic treated and untreated diabetic rats. Other researchers also found similar results after *H*. *rosa-sinensis* treatment, such as anti-implantation [51–52], abortion [53], and anti-fertility effects [54–55]. However, the implanted embryos from treated non-diabetic rats showed normal development. These findings contraindicate *H*. *rosa-sinensis* extract use during early pregnancy. In diabetic rats and their offspring, the plant extract showed beneficial effects on maternal reproductive performance. Our findings showed that STZ-induced diabetic rats had increases in both embryo loss and the incidence of fetuses classified as SPA, indicating intrauterine growth restriction, which is in agreement with the high proportion of fetal malformation due to hyperglycemia and/or oxidative stress previously reported [14,24]. After treatment with *H*. *rosa-sinensis* extract, there was a decreased rate of pre-implantation loss in diabetic rats and increased rate of fetuses classified as APA, demonstrating a beneficial effect of this plant extract that is potentially related to the improved lipid profile found in this specific group. However, treatment with *H*. *rosa-sinensis* did not reverse maternal diabetes-induced fetal abnormalities.

## Conclusion

The present study demonstrates beneficial effects of this plant extract in diabetic pregnant rats, in both mothers and their offspring, and no benefits were identified in non-diabetic rats. However, although these findings cannot be extrapolated to humans, they show that the indiscriminate intake of *H*. *rosa-sinensis* extract may be harmful to healthy individuals and its use should be completely avoided in pregnancy.
